# The Regulation of the Holy Hospital Hagios Panteleimon: Administrative and Ethical Framework under the Ottoman Hegemony

**DOI:** 10.7759/cureus.99311

**Published:** 2025-12-15

**Authors:** Christos Kazazis, Ioannis Nikolakakis, Nicholas Tentolouris, Ioannis Psycharis, Marianna Karamanou

**Affiliations:** 1 Department of History of Medicine and Medical Ethics, National and Kapodistrian University of Athens School of Medicine, Athens, GRC; 2 Department of Emergency Medicine, Tzaneio Prefecture General Hospital of Piraeus, Athens, GRC; 3 Department of Internal Medicine, Laiko Hospital, Athens, GRC; 4 Department of Economic and Regional Development, Panteion University of Social and Political Sciences, Athens, GRC

**Keywords:** administration board, head doctor, head nurse, hegemon, hegemony, hospital, ottoman, patients, regulation, samos

## Abstract

“Samos Holy Hospital Hagios Panteleimon” was founded on Samos Island, which at the time was part of the Ottoman Empire, in the second half of the 18^th ^century. It was not until the beginning of the second decade of the 20thcentury, just a few months before unification with Greece, that a Regulation of Procedure, embracing all aspects of its function and operations, was published by the Ottoman-appointed hegemonic regime in the local government gazette. Despite the Samian society’s gender and patient inequalities that are also reflected in its articles, this regulation was the first serious attempt in the hospital's history to establish a systematic approach to this institution’s mode of administration, staff selection, training, and duties, as well as an ethical frame for delivering care to all patients.

## Editorial

The island of Samos, situated in the eastern Aegean Sea, experienced a unique political and administrative trajectory during the nineteenth century. Following the Greek War of Independence (1821-1829), the island did not immediately join the newly founded Greek state but was instead organized as a semi-autonomous polity under Ottoman suzerainty, known as the Samos Hegemony (1834-1912) [1]. The Hegemony’s highest administrative authority was the Hegemon, or Prince, a Greek-speaking, Orthodox Christian citizen appointed by the Ottoman Porte. The Hegemon ruled along with locally elected representatives, who formed the local Parliament. The latter’s role was mainly to ratify any decree or other legal document issued by the former [2].

The hospital’s foundation and operation must be understood within the broader context of European and Ottoman public health reforms of the nineteenth century. Within the Ottoman Empire, the Tanzimat reforms (1839-1876) promoted modernization in administration and public institutions, including health services [3]. According to some researchers, these reforms were often used as a way to mitigate the dissent of specific populations within the empire [4].

A search was conducted in two main directions: Samos Island’s General State Archives and the Internet. In the latter, we searched for published papers using the keywords and key phrases “history of hospitals”, “male nurse”, “Head-Nurse”, “male and female nurse inequalities” and “patient care inequalities”. In the former, an extensive search was conducted in three archived collections: the Hegemonic Decrees from 1834 to 1912, the Hegemonic Government Gazette, with special emphasis given to finding material connected to the Samos Hospital establishment and mode of operation during the hegemonic period and the “Samian Printed One-sheets 1832-1915”, a three-volume printed collection published by Samos General State Archives, containing historical notes on the hegemonic regime, abstracts of all the Hegemonic Decrees and Hegemonic Circulars, as well as abstracts of Royal Decrees published by the Greek Administration during the first three years of unification of Samos Island with Greece.

The establishment of Samos Island Hospital

**Figure 1:**
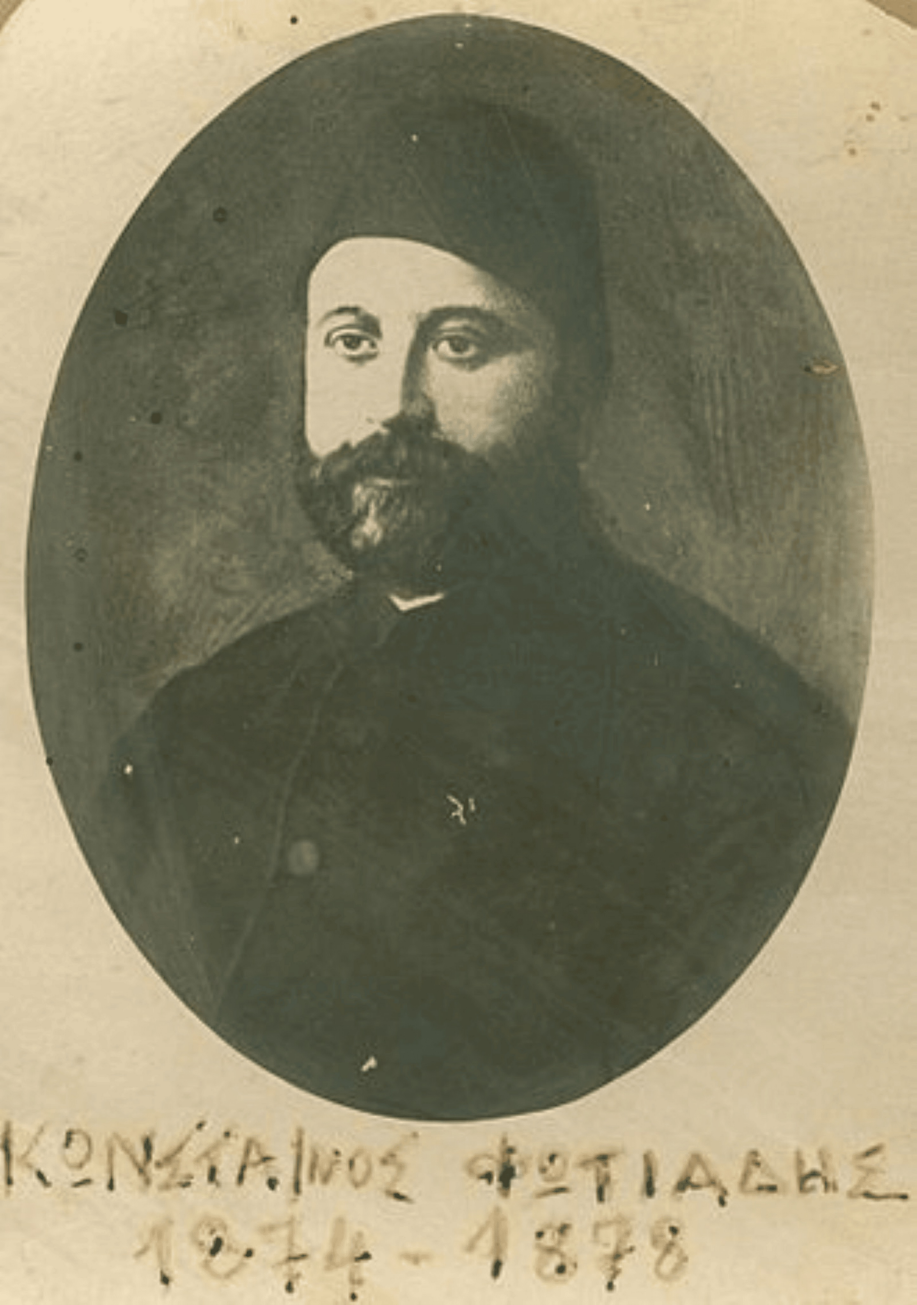
Konstantinos Fotiadis, Hegemon of Samos Island (1874-1878) Image in the public domain via Wikimedia Commons [8]

**Figure 2:**
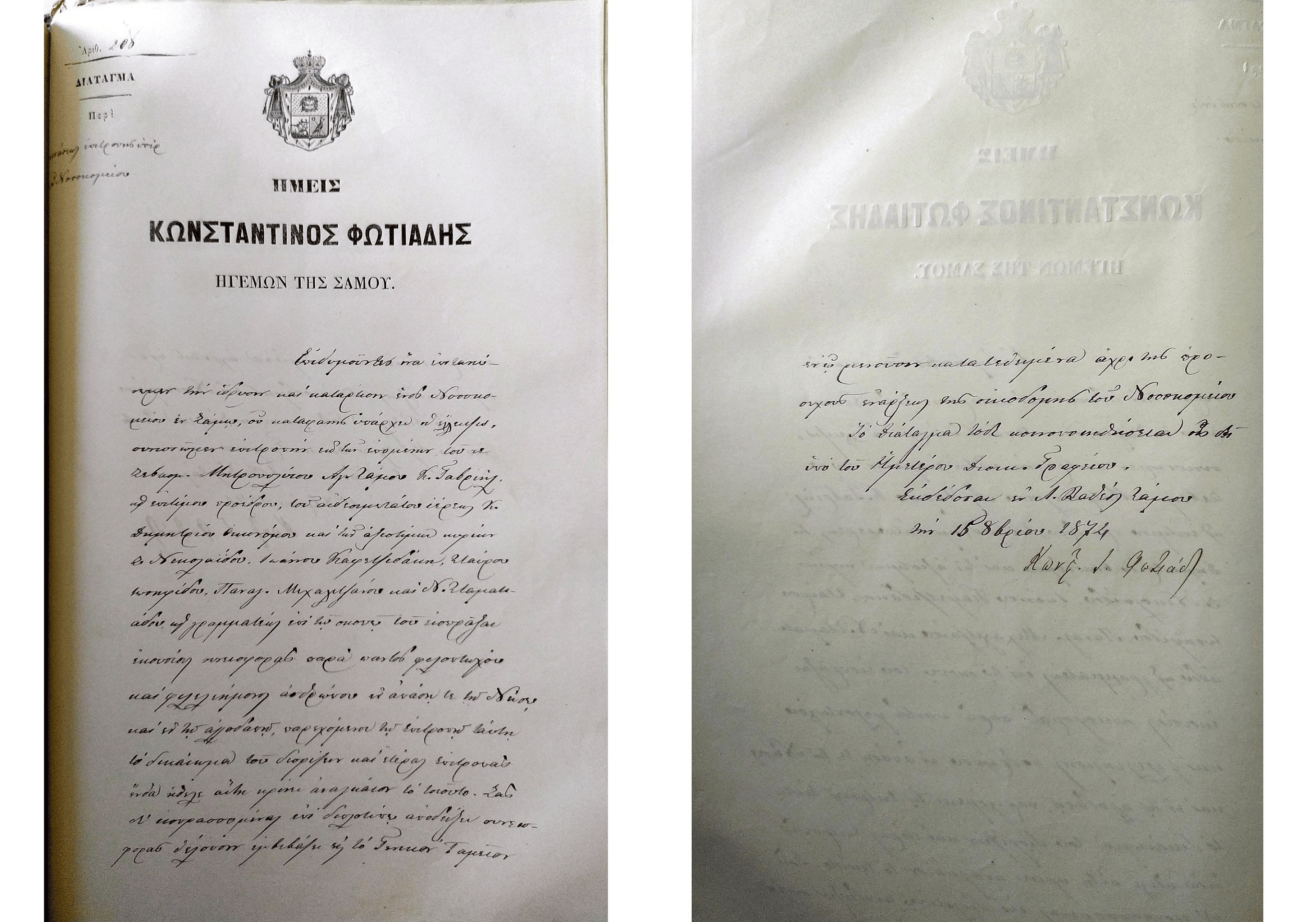
Hegemonic Decree 206 Published with permission from General State Archives, Samos Island, Greece [5].

Until the eighth decade of the 19th century, such an institution did not exist on Samos Island. The first attempt to raise funds for its construction was Hegemonic Decree number 206, issued on 16 October 1874 [5] and signed by the ruling Hegemon Konstantinos Fotiadis (Figure 1). In 1877, the same Hegemon signed Decree number 3, appointing a three-member committee to review the operating procedures of other hospitals and develop the local hospital’s procedural regulations while also considering the specific needs and resources of the local community (Figure 2) [6]. Decree number 121, issued in 1899 by the Hegemon Konstantinos Vaianis, attempts to establish some order in the hospital’s function and internal procedures [7]. It was not until the end of the hegemonic regime that a serious attempt was made to establish a comprehensive set of rules governing all in-hospital operations.

The 1912 regulation of procedure of "Samos Holy Hospital"

On the second of May 1912, at the Port of Vathi, the "Samos Holy Hospital Hagios Panteleimon” Regulation of Procedure was published in the Hegemony’s Government Gazette, signed by Hegemon Grigorios Vegleris [9]. It consisted of 92 articles, the vast majority of which were grouped in 16 chapters, covering every aspect of the hospital's functional requirements, with emphasis given to issues concerning administration, financing, doctors’ and nurses’ duties, as well as patients’ standards of well-being and ethical treatment. In this paper, we will present and discuss Articles 1 to 58, which concern the hospital's administration, staff selection and duties, and provisions on patients’ treatment and medical ethics.

Administration (Articles 1 to 10)

The first two articles state that the hospital is under direct Government control since, after parliamentary proposal, the Hegemon appoints a six-member administrative board called “Ephor`ia” (Ephorate). It consists of three men, one of whom is a medical doctor, and three women “of good reputation and literate”. One board member is appointed as the hospital's treasurer.

Articles 3 to 10 are grouped under the first section, “Duties of Ephor`ia.” Article 3 defines its aims, which are the hospital's material development and moral advancement, so as the latter will become not only financially independent but also a center of charity in the area. Nevertheless, it is clarified that the board cannot interfere with the duties of the medical or nursing services, leaving the hospital's head doctor and head nurse solely responsible for them. According to Article 4, board meetings must be held once or twice a month, minutes of which must be recorded in detail, containing every thought relative to the institution’s interests. When issues relating to medical or nursing services are under discussion, the head doctor or head nurse must be present, though without voting rights on the decisions taken. Article 5 requires at least four of the board members’ signatures and the hospital's seal for the validity of any document issued by the former. Article 6 holds the board responsible for the management or loss of the institution’s property, the collection of any debts towards the hospital, as well as the duty to buy the Hegemony’s bonds. Furthermore, the treasurer is solely responsible for any cash entrusted to him. Besides, the treasurer, as stated in Article 8, collects every kind of financial income by using duplicate receipts initiated by the government's General Accounting Office. Acting on the basis of payment lists signed by the administrative board, the treasurer is responsible for executing all financial transactions and presenting monthly reports on the hospital's income and expenditures. He also maintains the required accounting ledgers, which must be certified by the government's General Accounting Office. Last but not least, the treasurer is responsible for the disposal of the hospital's assets in accordance with the administrative board’s decisions, the approval of the hegemonic administration, and the laws of the state. Article 9 provides further protection of the hospital's property by banning every kind of sale or expropriation without the approval of the Hegemonic Administration. The same approval is needed, according to Article 7, to validate the board’s proposals on each staff appointment, including the hospital's priest, head doctor, and pharmacist. Article 10 demands that the administrative board submit annual reports to the Hegemonic Administration, which should include incomes and expenses as well as staff behavior and its performance improvement or deterioration. In the same article, it is clearly stated that no reforms from the board can be made in the hospital without prior “scientific review” and approval by the state's administration.

Female Head Nurse’s Duties (Articles 11 to 13)

At least a six-year period of “honorable service” to recognized healthcare facilities is considered a prerequisite to qualify for this position, according to Article 11. Her duties, described in Articles 11 and 12, were multiple and crucial for the hospital’s function, the building’s safety, and patients’ well-being.

First of all, she was responsible for the allocation of any service to the hospital's staff and apprentices, who were under her direct command.

Secondly, it was her duty to execute the board’s commands and, in every meeting, to report on anything that took place in the institution.

Thirdly, she had to procure in time food and any necessary provisions after the board’s corresponding tender offer, as well as to take delivery of any materials and provisions entering the Institution.

Furthermore, patients’ hygiene, inspection of the hospital's premises, and inspection “with her own eyes” at least once during the night, at random hours, of patients’ wards, to make certain that they are clean and in order were another everyday obligation she had to fulfill.

In addition, keeping the keys of the hospital's main entrances and warehouses and being the “vigilant guard” of the hospital's regulation of internal operation, with emphasis on strict implementation of a moral code of behavior among staff and patients, were also among her main duties.

Last but not least, Article 13 foresees the provision of shelter and in-hospital care for the female head nurse, while her payment is specified by the board and registered in the state budget.

Medical Service and Hospital Doctor's Duties (Articles 14 to 19)

Provision is made for only one hospital doctor, who can, if needed, hire an assistant (Article 14). According to Article 15, the head doctor makes his rounds in patients’ wards at 7.00 pm during summer months and at 8.00 pm during winter, listening to either the female nurse’s or the male head nurse's report (see also Article 22) and instructing them accordingly on patients’ treatment and diet, signing at the same time the hospital formulary. After completion of hospital rounds, the head doctor is expected to examine and provide consultation to outpatients as well as to distribute free medicine to indigent patients. Article 16 is dedicated to the hospital doctor's obligation to additionally visit seriously ill patients during the evening and whenever he feels it necessary. He also must respond to every emergency call and is generally held responsible for every patient. The head doctor reserves the right to form a medical council consisting of medical doctors practicing in the area of the Port of Vathi in order to examine cases of seriously ill patients according to Article 17. Article 18 states that the nurse service is under the head doctor's commands, and only he and the female head nurse can intervene in patients’ treatment and diet. Furthermore, the head doctor is required to keep two books. The first one contains information on patients’ admission, discharge, or death, while the second holds records of any medicine given to patients on a daily basis (Article 19).

Duties of the Hospital’s Pharmacist (Articles 20 to 22)

To be eligible for this post, the candidate must meet three prerequisites stated in Article 20, namely, to hold a degree from a recognized pharmaceutical school, to have at least 10 years of service as a pharmacist in a recognized pharmacy, and to demonstrate an understanding of hospital procedures. Furthermore, Article 21 describes the pharmacists’ in-hospital duties, which consist of the good and according to the rules of the profession, maintenance of the medicinal products as well as the Pharmacy’s utensils, execution of prescriptions, labeling each medicine with the patient’s room and bed number, as well as prescribing the mode of administration, following the Hospital Doctor’s instructions. The Pharmacist is also responsible for maintaining records of any executed prescription, whether from a regular or extra-regular formulary, and for keeping the prototypes until audit, informing in time the administrative board of the Pharmacy’s needs. The Pharmacist stays, if necessary, inside the Hospital and exits at specific hours.

Article 22, even though it is included in the pharmacist's section, actually concerns duties of the male head nurse, which are the “unfailing implementation” of the hospital doctor's instructions, the execution of any duty that is assigned to him by the former, the assistance of the ward nurse, the written invitation of the hospital doctor for the daily rounds and, consequently, the duty of following him in the wards.

Hospital Staff (Articles 23 to 32)

This section refers to the qualifications, training, and duties of female hospital nurses. Qualifications for admittance are described in detail in Articles 23 and 24. In order to be accepted for training, a woman had to be between 20 and 45 years old, free or widowed, and without any family obligations, as well as of good health, with high moral and philanthropic standards, without any character defects, and able to read and write. Candidate nurses are considered qualified by the female head nurse after a two-month training period. However, according to Article 25, further theoretical and practical training is carried out by the female and male head nurses whenever the candidates feel ready to nurse patients. Except for the usual medical and general care for the patient and the wards, they are expected “to confiscate” any food that is supplied from visitors to patients, without the Hospital Doctor’s approval, and to return confiscated items to visitors once they exit the establishment. In addition they are expected to carry and distribute patients’ food, taking extra care not to spill it on beds or the floor, and in case of such an event to clean it immediately and diligently. They must also prevent any exchange or sale of food among patients and they have to report to the male Head Nurse any patient who leaves the wards without the Doctor’s authorization. Furthermore, they report to the male Head-Nurse any deterioration in patients’ health status, especially cases of hemorrhage, spasms, dyspnea, serious weakness, and vomiting, and they must avoid shouting and making noise, thus disturbing patients’ sleep. Last but not least, they have the duty to verify the wards’ integrity and to report to the Head-Nurse any damages that come to their attention (Articles 26 to 31).

Article 32 determines that nurses receive food from the hospital, wear the same uniform, and exit the hospital after approval from the female head nurse.

Patient Provisions

General provisions (Articles 33 to 45): According to Article 33, patients from any nationality or religion are accepted in the hospital, except, as stated in Article 34, patients suffering from first- or second-stage tuberculosis, those with acute infectious or incurable diseases, patients with psychiatric conditions, and patients diagnosed with venereal diseases.

Article 35 foresees that if the patient is indigent, he or she has to demonstrate the corresponding certificate, issued by the mayor of his or her municipality, along with a “scientist doctor’s” report on his or her nature of disease. A fine ranging from 100 to 200 Ottoman Groschen (γρόσια) is imposed on patients in cases of forged certificates, according to the state’s criminal law. The same fine can be imposed on the head doctor or the head nurse for not reporting such cases directly to the government in order for the latter to take measures against the mayor, who issued the false certificate, as well as to charge him with the expenses made by the patient. A certificate is not needed upon admittance, only in case of a sudden accident. Nevertheless, the mayor has to issue one in the next twenty-four hours.

Article 39 states that once admitted, no visits can be made to the patients without the head doctor's prior consent.

Articles 40 and 41 arrange matters concerning incoming patients’ clothing and valuables.

Article 42 guarantees a patient's right to refuse a painful operation, and in the case of a mentally incapable patient, his or her father or closest relatives have to provide consent for the procedure.

Articles 43 to 45 clarify matters around patients’ hospital exit, which is prohibited without the head doctor's prior consent and is allowed only in cases of urgent matters that require the patient’s attention. Failure to adhere to any of the hospital regulation provisions gives the right to the head doctor to expel the patient from the institution.

Patients in Danger (Articles 46 to 53)

Articles 46 to 48 refer to the female head nurse's duty to inform gravely ill patients’ relatives accordingly, who can visit the latter “without shouting and crying.” If the patient wishes, she can arrange a visit by a priest and, according to every patient’s religious dogma, inform the corresponding higher religious authorities.

Article 49 states that the bed of such patients should be placed in a “movable enclosure.”

Articles 50 to 53 describe the procedure that should be followed after the death of a patient: The male head nurse reports the death to the hospital doctor. The latter confirms the death and issues a certificate for the mayor so he can issue a burial permit. The doctor also signs the death certificate, marking the day and time of death, and delivers it for further processing to the corresponding registry. If the deceased patient’s relatives do not appear, the female head nurse arranges the funeral at the hospital's expense. The patient’s bed linen is “immediately” replaced, and the bed itself is disinfected. In case this is not enough, the bed mattress is incinerated.

Paid-up Patients’ Section (Articles 54 to 58)

Articles 54 and 55 foresee that wealthier patients “wishing” to be admitted to the hospital can do so after paying a fee in advance and every two weeks afterward at the hospital's cash desk. No reimbursement is made if the patient exits the hospital before the end of the two-week period without the doctor's consent. Article 56 provides this category of patients the right to invite, at their own expense, doctors for consultation who do not work in the hospital. In this case, the hospital doctor always takes part in the consultation, also at the patient’s expense. Article 57 states that this group of patients’ diet is prescribed daily by the hospital doctor. Relatives of wealthy patients can visit them three times a week at certain hours according to Article 58.

This is the first Regulation of Procedure of Samos Hospital, which addresses virtually every aspect of its everyday function. There is no doubt that the administrative board holds a vast range of powers, ranging from hospital financial support to staff selection and future planning of the institution’s further development. The fact that the board consists of three men and three women is rather surprising and groundbreaking, since Samos was, at that time, a male-dominated society where sex inequalities were embedded in legislation and everyday life.

It is undeniable that the presence of a head nurse in a hospital is necessary in preventing in-hospital conflicts, which can affect the quality of care [10]. This is also reflected in the duties of the female head nurse, as stated in Articles 26 to 32 of this regulation. However, contrary to the board’s synthesis, selection of the female and male head nurses poses some critical questions on sex inequalities in this particular workplace during that period. While the regulation contains clear and very demanding criteria for the selection of the female head nurse, this is not the case for the male one, where no specific selection criteria are registered. The same goes for the former’s duties, which are much more clarified and demanding than the latter’s duties.

Despite this fact, the male head nurse seems to be, from an administrative point of view, closer to the head doctor than the female one. It is of note that the male head nurse is the only one responsible for reporting to the head doctor a patient’s death, while the female head nurse has the duty to arrange the funeral of patients whose relatives do not appear to receive the corpse. Nevertheless, some authors argue that in practice, the opposite was true, with male nurses, historically, facing prejudice and unfair professional treatment from their female counterparts and the state [11]. This could provide a possible explanation for the fact that in the present regulation, there is no provision for accepting male nurses for training and work in the hospital, except for the male head nurse, for whom, as mentioned already, no specific qualifications and training are foreseen.

Article 23 constitutes another paradigm of gender inequality by demanding the future nurses to be either free or widowed and without any family obligations, whereas no such requirements are made for the rest of the hospital staff.

There is no doubt that by accepting patients for treatment in “Samos Holy Hospital”, regardless of their race, religion, or financial status, the Samian society is moving towards the right direction by the end of the 19th century. Despite this progress, inequalities in patient care are still evident in this regulation. First of all, a significant number of patients with life-threatening medical conditions are banned from hospital access, as stated in Article 34, and there is no evidence found in the local State Archives of the procedures followed, if any, in case they needed hospital care. Secondly, there is a strict protocol of accepting indigent patients for treatment, which is understandable up to a certain point, given the fact that this group of patients was treated for free, and cases of document fraud to avoid paying treatment expenses could ensue. However, the mandatory requirement for an indigent patient to carry an external doctor’s note explaining the need for hospitalization, while this is not explicitly required for patients paying their fees, who can demand admission and treatment at their own will, is another sign of unequal access to care. Furthermore, paid-up patients reserve the right to request a medical consultation from the hospital and private medical doctors, which was not possible for an indigent patient, leaving this right to the head doctor alone. Last but not least, a specific provision for everyday instructions from the head doctor on paying patients’ diet, as well as a much more flexible visiting regime from their relatives compared to that of the indigent ones, reflects another important inequality in patient care.

Having mentioned that, Articles 42, 46, 47, and 48 represent a safeguard for patients’ right to consent to any in-hospital medical intervention, and they formulate a legal framework for family involvement in treatment decision-making for those who are mentally unable to do so. Besides provisions in these articles aiming to respect the spiritual wishes and religious needs, especially of those who are seriously ill, are comparable with contemporary views on cultural competence that health providers and organizations are expected to deliver [12].

It is undeniable that the 1912 Regulation of Procedure of the Samos Holy Hospital represents a landmark in the institutional history of healthcare within the Samos Hegemony. As the first comprehensive framework governing all aspects of hospital administration, staff duties, and patient care, it demonstrates a remarkable attempt to align a provincial institution with broader trends of medical modernization in the late Ottoman and European context. The inclusion of both men and women on the administrative board, as well as the detailed definition of the female head nurse's responsibilities, marks innovative features for their time, even if gender inequalities remain evident in the asymmetrical treatment of male and female staff. Likewise, while the regulation promoted equality by granting access to patients irrespective of race and religion, distinctions between indigent and paying patients reveal persistent disparities in the quality and scope of care provided. Provisions concerning informed consent, patient rights, and respect for spiritual and religious needs, however, signal an early awareness of ethical standards in medicine. Taken together, this regulation reflects both the limitations of a society still shaped by inequality and the progressive aspirations of an administration striving to institutionalize accountability, professionalism, and patient-centered care on a remote Aegean island at the dawn of the 20th century.
